# Avian Influenza Risk Environment: Live Bird Commodity Chains in Chattogram, Bangladesh

**DOI:** 10.3389/fvets.2021.694753

**Published:** 2021-09-20

**Authors:** Erling Høg, Guillaume Fournié, Md. Ahasanul Hoque, Rashed Mahmud, Dirk U. Pfeiffer, Tony Barnett

**Affiliations:** ^1^Department of Global Health and Development, London School of Hygiene and Tropical Medicine, London, United Kingdom; ^2^Department of Pathobiology and Population Sciences, Veterinary Epidemiology, Economics and Public Health, The Royal Veterinary College, London, United Kingdom; ^3^Department of Medicine and Surgery, Faculty of Veterinary Medicine, Chattogram Veterinary and Animal Sciences University, Chattogram, Bangladesh; ^4^College of Veterinary Medicine and Life Sciences, City University of Hong Kong, Kowloon, Hong Kong, SAR China; ^5^Humanitarian and Conflict Response Institute, University of Manchester, Manchester, United Kingdom; ^6^Firoz Lalji Institute for Africa, London School of Economics, London, United Kingdom

**Keywords:** Bangladesh, avian influenza, commodity chains, risk environment, agency-structure, anthropology

## Abstract

In this paper, we identify behaviours in live bird commodity chains in Chattogram, Bangladesh, which may influence the risk of pathogen emergence and transmission: the nature of poultry trade, value appropriation and selling sick or infected birds. Examining the reasons why actors engage in these behaviours, we emphasise the politics of constraints within a context of real-world decisions, governed by existential and pragmatic agency. Focusing on *contact zones* and *entanglement*, analysing *patron-client relationships* and *precarious circumstances*, we argue that agency and structure specific to the Bangladeshi context produce a *risk environment*. Structural constraints may reinforce risky occupational practises and limit individual agency. Structural constraints need to be addressed in order to tackle animal and zoonotic disease risk along live animal commodity chains.

## Introduction

Anthropology of poultry commodity chains with a view to understanding behaviours and practices that may influence the spread of bird flu viruses remains a novelty. Anthropological studies of avian flu biosecurity, risk and practises in Asia, have emphasised *difference, variety* and *gaps*. Anthropological studies of *different logics of biosecurity and care* have compared the opinions of backyard farmers and commercial farmers in Indonesia (1) and farms and laboratories in Hong Kong (2). Anthropological studies focused on *variety of biosecurity* have compared corporate, state and systems security with farm biosecurity in Indonesia (3) and how global-state-society relations affect bird flu management in Vietnam (4). Porter also showed how risk perception may vary significantly within a local population in Vietnam, and how divergent risk maps, made by poultry farmers and health workers, represent the wider difficulties in how to define and manage avian influenza risks (5). An anthropological study of the reorganization of health system and health management in Hong Kong after the first outbreaks in 1997 emphasised both difference and omission, as this was being done *without using notions such as ‘Asian culture' and ‘Asian ecology'*, ignoring the conditions and context behind bird diseases, showing the gaps between a scientifically and institutionally portrayed reality and a lived reality (6). In other words, from our perspective, in reorganizing the health system, they should have described, understood and taken action upon the complex entanglements of actors in poultry transactions. In a similar vein, an anthropologist followed transnational avian influenza scientists and poultry breeders in China and showed how farmer practises displace scientific research, as farmers refuse to accept and act on the category distinction between “wild” and “domestic” (7).

Epidemiological studies have evaluated how live poultry movement and trading practises influence the spread of avian influenza viruses in Cambodia [e.g., (8)], Vietnam [e.g., (9)], Thailand [e.g., (10)] and China [e.g., (11)]. Focusing on the structure of poultry trading networks and the structure of contact between individuals, these cross-sectional and longitudinal studies have used descriptive surveys and social network analysis. A common finding points to how poultry farms and live bird markets are highly connected, indicating potential routes for the spread of infection.

Here we identify those behaviours at nodes between production sites, market maker scenes and final outlets in Chattogram^1^ Division that may influence the risk of pathogen transmission. Our focus is not on behaviours in the conventional sense, like the use of personal protective equipment, hand washing and biosecurity practises, but unrecognised risks which are generated through live poultry trade along the commodity transaction chain. We focus on the day-to-day pragmatics of trading behaviours towards gaining a deeper understanding of underlying beliefs and rationalities: how and why people conduct themselves in their everyday “taken for granted” working lives. To do this, we see and problematize everyday life as novel, recording its structures, examining in context how the probability of emergence is amplified through inappropriate human-animal contact or entanglement (12) within these pragmatic, livelihood related, encounters.

### Risk Environment

We argue that the diversified, uncontrolled poultry trade, value appropriation and the trade in sick or infected birds can be explained by the conditions within which behaviours occur, which has been termed a *risk environment* (13). A risk environment for exposure to infectious diseases delineates social, physical, economic and policy contexts in which a variety of factors exogenous to the individual interact to promote the adoption of risky practises, increasing the chance of harm occurring. Such focus on risk environment stems from the idea that social structures determine individual practise, simultaneously recognising that a risk environment is a product of the *inseparable interplay* between micro-, meso- and macro-level factors. In other words, there can be several types of interacting and overlapping risk environments in a given context. In this respect, the processes are multidimensional in space, time and scale (14). A risk environment specifies how cultural and social structures determine individual and group action in unintentional and indirect ways, normalising certain responses to situations and events (15). Therefore, occupational practices may become legitimate social practises, as predicted by sociological constraint and structuration theories (16). Shared knowledge and practises can be seen as practical consciousness; taken for granted survival strategies in the context of limited resources and an abundance of constraints. Such everyday rules may be incongruent with the prescriptive laws of governments, religions and other ethical systems, but become legitimate, acceptable practices, if the food supply chain is to continue to meet demand and people are to be able to maintain their livelihoods. While on an everyday basis these practises concern business resilience and continuity, they may increase the probability of pathogenic spread and threaten public health. Considered anthropologically, everyday practises have *purposes, reasons* and *intentions*, analysed by how individuals exercise different forms of agency, depending on their temporal orientation – immediate, short or long-term. Agency is seen as historically contingent, graduated, and distributed, emphasising the predominance of *existential* and *pragmatic* agency (17, 18).

Our focus on risk environment provides contextualisation of risky behaviours, caused by factors related to agency and structure. We apply the concepts of agency and structure from practise theory [e.g., (16, 19)]. *Agency* concerns the ability to “make things happen,” the capacity of an individual or group – professions, governments and organizations – to act against constraining structures. *Structure* describes the conglomerate of rules, resources, forms of domination and power (16).

### Political and Economic Constraints

Since 1960, the population of Bangladesh has increased 3.4 times from 48 to 163 million (2019), while the population of chickens and ducks has increased 13.6 times from 23 to 338 million (2,108) (20–22). About five million people take care of the birds (23). Since the first reported avian influenza H5N1 outbreak in Bangladesh in 2007, 555 outbreaks have been reported (24).

The occupational practises occur in the specific political and economic context of Bangladesh. The Bangladeshi economy has improved significantly in recent decades, the growth driven by increases in agriculture, including livestock, construction, manufacturing, the textile industry, export and remittance (25, 26). However, since its rapid expansion with new poultry types in the 1990s, the live bird sector^2^ has operated under the rules, resources and administrative politics of Bangladesh. These are structural constraints. From the perspective of anthropology of the state, this can be seen as a *structural effect* (27), pointing to current practises as a consequence not of the exercise of power, but of state powerlessness. Routine active surveillance of avian influenza remains limited, due to insufficient infrastructure, human and financial resources in the livestock department. Therefore, the department relies on passive surveillance, awaiting poultry mortality reports from the farmers. In 2009, the Ministry of Fisheries and Livestock emphasised how the live bird sector evolved and continues to operate without maintaining minimum biosecurity standards that would reduce the risk of disease or pathogen transmission (28). By 2020, a new policy for the trade in live birds has not yet been developed and implemented, partly due to the government according low priority to avian influenza as compared to other threats and crises (29).

Unstable commodity prices can be explained in two ways: Larger socio-political structures determine the price fluctuations and the business actors seek to maximise their profit. This is analysed in terms of *price volatility* and in the results section as *patron-client relationships* (relationships wherein processes of *value appropriation, trust, mistrust* and *risk* occur).

#### Price Volatility

Chicks, feed, medicines and live birds are liable to rapid and unpredictable price changes, dependent on *the price of raw materials, absence of government price regulation, changing taxation rules* and *illegitimate price increase*. First, production costs increase, when the price of raw material increases on the international market. For example, feed companies sell their products at a higher price, when the price of feed ingredients like soybeans and maize increases.

Second, the government has been unable to regulate commodity prices. The role of the Trading Corporation of Bangladesh, TCB, as commodity price regulator has eroded under the growing dominance of free market policies. Public demand to make the TCB effective at the domestic level has met resistance from the business community syndicate and business lobbies within the government, including disputes between the four ministries of *Commerce*; *Food and Disaster Management*; *Finance*, and *Home Affairs*, which impede emergence of political consensus (30). A Commodities Exchange has been proposed as an efficient way to counter price volatility on the Bangladeshi market with the intention that prices would stabilise. This would remedy uncoordinated supply chains, unfair prices faced by the farmers and the many speculators between farmers and consumers (31). Such stability would partly counter risky occupational practises, thus reducing transfer of potentially contaminated commodities. Farmers would be able to plan ahead, knowing about future prices. It would reduce structural constraints and increase their agency and preferential choices. But the implementation of a Commodities Exchange has been unsuccessful for the same reasons as the failure of the trading corporation.

Third, the government has introduced, withdrawn and reintroduced various tax regimes since the 1990s. In 1991, the Tax Act introduced a Value Added Tax of up to 15 per cent on goods and services with exemptions for certain essential commodities and services: consumables, agricultural inputs and products, animal products, financial activities, social welfare, transport services, *the poultry sector* and more (32). However, periodic withdrawal of such exemption has created waves of taxation rules and confusion. This has been depicted as “tax bargaining,” within the broader Bangladeshi political economy, characterised by deep rooted informal institutions, norms and networks, underpinning the combination of weak governance and strong economic growth. This has made the existing system attractive to powerful economic and political interests, providing fertile ground for patronage politics. In this way, the tax system has been constantly contested and renegotiated, liable to rapid and *unpredictable* price changes.

Fourth, domestic market prices are affected by profiteering trade syndicates, price manipulation, corruption, as well as extortion and unofficial payments in the transportation, distribution and marketing channels of food. This creates an artificial crisis of essential commodities (30, 33). Such illegitimate price increase is one of the adverse consequences of a supposedly “open” market system.

## Methods

Our focus provides explanation and contextualisation of risky behaviours, caused by factors related to agency and structure. The call to understand *why* actors engage in risky behaviours is not new in interdisciplinary collaboration between anthropologists and epidemiologists (34–37). The *why* question remains a classical anthropological method, as opposed to epidemiology, which focuses on identification of behaviours through *who, when, where*, and *how* questions (35). A focus on behavioural causes, including factors related to agency and structure, points to the anthropological emphasis on disease ecology, scrutinising the circumstances associated with the emergence and outbreak of infectious diseases: historical, political, economic, social and cultural context (37). Epidemiology often lacks such contextual understanding (36).

### How, What, and Why

Anthropology offers qualitative methods and analytical tools with a focus on description and interpretation of context and experience, based on observation and open-ended questions. Multi-sited fieldwork of people, their connexions and relationships (38) and grand and mini tour observations (39), asking classical ethnographic *how, what*, and *why* questions, was conducted between February 2017 and February 2018. This necessitated cumulative snowball sampling, semi-structured and ethnographic interviews, participant observation and qualitative surveys (40).

Through an inductive cumulative research process, we identified eight specific roles between production sites and outlets: *farmer, feed dealer*, first *line middleman*, second *line middleman, market middleman, market broker, wholesaler* and *retailer*. Initially, we were familiar with farmers, feed dealers, middlemen and market wholesalers and retailers, but through our fieldwork discovered the differentiation between three kinds of middlemen and also identified the market broker, distinct from wholesalers and retailer.

The study had three main analytical incentives. First, we targeted *decision making processes* and *hierarchical relations* in the poultry trading network: how buying and selling chicks, feed and medicines influence the poultry trade. This allowed an investigation of the hierarchical relations in the business networks between them, identifying the patterns and variations of live bird trade and exploring how credit and debt relations influence these hierarchies. For comparative purposes, we asked the same questions to farmers, feed dealers, middlemen, wholesalers and retailers: about their business relations, how they negotiate the price of poultry, how they collect information about the daily poultry market price and their responses to disease outbreaks (27 questions). Second, we targeted *trading patterns, price variation, business relations* and *responses to infectious disease outbreaks* among small- medium- and large-scale cash and credit farmers^3^ in nine Chattogram sub-districts (21 questions). Farmers were randomly selected within each subdistrict: (1) near the main town, (2) semi remote farms, and (3) remote farms among poor, middle class and rich farmers. Third, we examined *commission and profit* for day-old chicks, feed and medicines among feed dealers in five Chattogram sub-districts. The focus was on the interactions between hatcheries, feed millers, drug companies, feed dealers and farmers.

At the outset, we planned the number of interviews to be conducted. For the decision making and hierarchical relations research theme, we planned and conducted 30 interviews. For the farm study we planned 120 interviews, but due to heavy weather conditions during the rainy season, making it impossible to reach farmer destinations, we managed to conduct 80 interviews. Sometimes it was difficult to locate the farms. Some farmers were unwilling to give an interview. For the feed dealer study, we had planned 50 interviews, but due to weather conditions and the fact it was often difficult to organise an interview with this type of actor, we managed to conduct 38 interviews (12 questions). In total, 148 interviews were conducted, each lasting between 1 and 3 h^4^.

For the grand tours, an observational checklist was used for descriptive observations, concerning *space, actor, activity, object, act, event, time, goal*, and *feelings*. These dimensions serve as guides for the participant observer (39). For every field site visit, the aim was to identify and describe its major features (a feed dealer shop, a poultry farm, the business environment of a middleman and a market vendor). Mini tour questions were then asked. For example, after describing a feed dealer's office, mini tour questions were about relevant details related to decision-making processes. For example: *Tell me about how you keep records of your trade in poultry*.

All interviews were conducted in Bengali and translated into English by a native Bangla speaker. Field notes, interviews and observations were organised, compared, coded and analysed, using a software programme for qualitative research.

### The Actors

This study examines three commodity chains between farmers and consumers: *white broilers, Sonali coloured broilers*, and *spent hens*. Breeds of white feathered broilers include Hybro-PN, Hubbard Classic, Cobb 500, Hybro-PG and Ross. White broilers grow quickly, compared to other meat producing birds. *Sonali* is a cross-breed between Rhode Island Red (RIR) cocks and Fayoumi hens. There has been an increasing demand for such coloured birds and Sonali adapt well to the climate and require less care and attention than other breeds (41). Layer farmers operate under intensive management practises for commercial egg production. They sell spent hens, those no longer able to lay.

**Figure 1:**
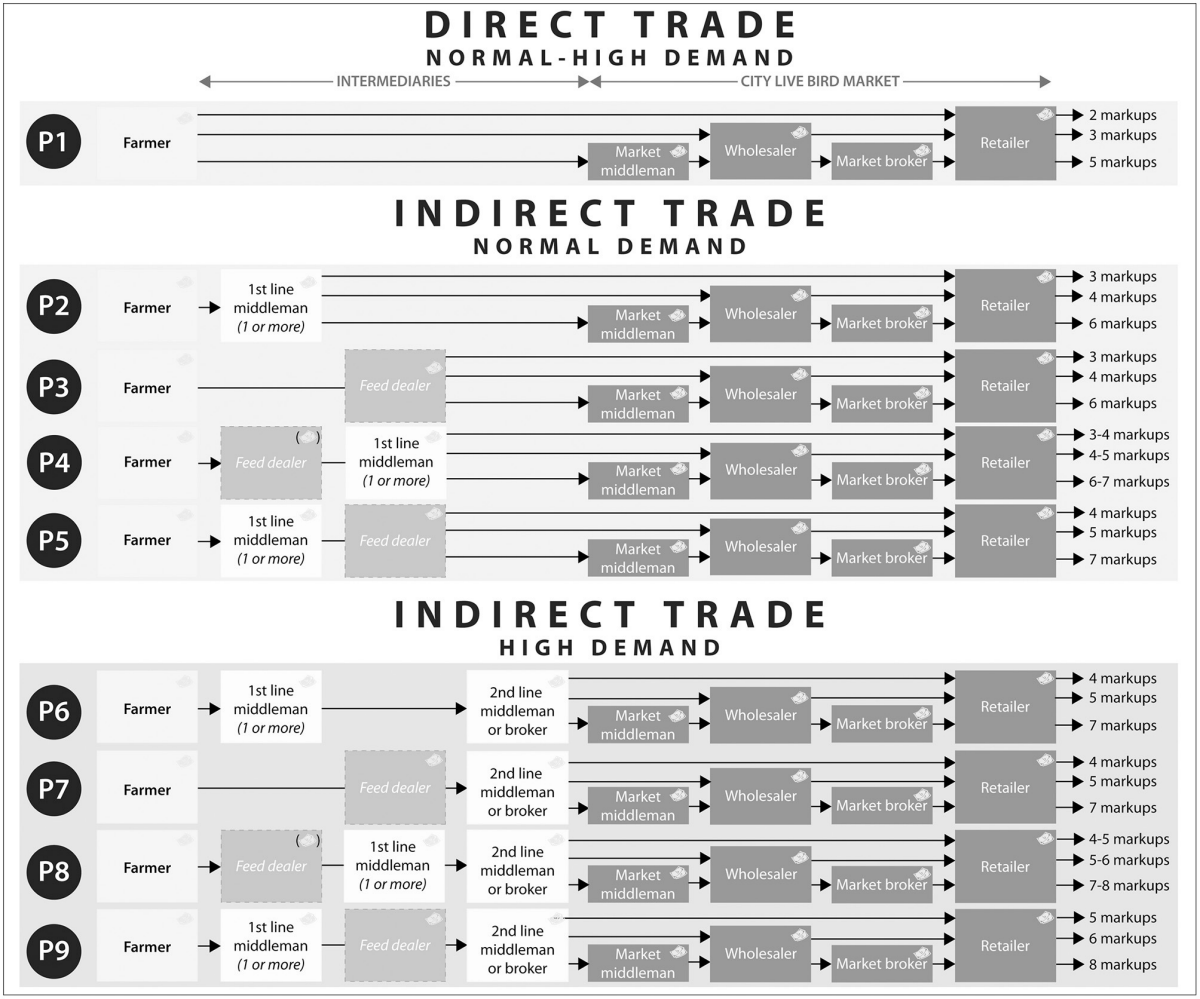
Poultry commodity chains in Chattogram, Bangladesh. Trading patterns P4 and P8: Feed dealers may or may not ask for a fee, when they help the farmers getting in contact with first line middlemen (money icon in parentheses).

Poultry commodity chains involve *producers* (cash farmers and credit farmers), *field traders* (feed dealers, first- and second-line middlemen, middlemen employees and brokers) and *market traders* (wholesalers, retailers, middlemen and brokers). Transactions from farm to market vendors occur in variable combinations and sequences (see Figure 1).

Farmers buy chicks, feed and medicines from feed dealers. *Cash farmers* buy these goods in cash, while *credit farmers* buy them on credit. There are different types of traders: *market middlemen, market brokers*, first *line middlemen*, second *line middlemen*, and *field brokers*. *Feed dealers* act as second party agents, dealing in key agricultural commodities: feed and medicines for poultry, fish and cattle; day-old chicks and poultry feeders and drinkers. They are *market makers*, known in the banking world as key commodity buyers and sellers [(42), p. 127], providing liquidity through credit arrangements and thus, at a fundamental level, enabling the markets to come into existence (43). Big and small *middlemen* act as intermediary traders between farmers and markets. First *line middlemen* operate during normal poultry demand. Second *line middlemen* enter the scene during high demand. Feed dealers and most big middlemen rarely touch or transport poultry. Feed dealers stay in their shops and big middlemen mostly operate from home or from teahouses. Brokers play a minor role in the trade of poultry. In contrast to poultry middlemen, brokers work in various businesses dealing in all kinds of commodities. They put buyers and sellers in contact, taking a commission for this service. *Small middlemen, middleman employees*, and *feed dealer employees* collect the birds at the farm gate in trucks and deliver them to markets. *Wholesalers, retailers, market middlemen*, and *market brokers* operate within the markets.

## Results

### Contact Zones, Entanglement, and Contagion

The encounters between people, live birds and viruses may constitute sites of avian influenza risk. Think of these encounters as contact zones that may lead to human-animal entanglement, an intermix of bird origins, bird types, people, precarity and pathogens. A contact zone represents sites of encounter and contagion (44) between humans and birds and between birds. Entanglement constitutes fundamental characteristics of disease emergence and ecology, which points to critical aspects of missing disease prevention and control, such as contact and disease tracing, biosecurity and trade configuration (45).

We emphasise six such encounters along the commodity chain and in markets.

***Human exchange:*** heterogeneous, diversified networks of commodity flows and exchange between production sites and outlets without a clear trade command hierarchy;

***Market destinations:*** the potential high number of market destinations for birds from a particular farm;

***Mixing of the same bird type:*** the potential high number of farms, from which a particular bird type originates at the individual market vendor level;

***Mixing of different bird types:*** several bird types come into close contact in the markets;

***Trade between markets:*** trade between markets is common;

***Mixing of different animals and commodities:*** live birds are mixed with other live animals and other commodities in the markets.

Trader roles vary: they may change, depending on circumstances, reflecting a fluid set of roles and relationships. Indeed, there are clear roles (e.g., farmer, feed dealer, wholesaler), but there are also shifting roles beyond exclusive categories. Traditional roles in live bird trade do not follow simple logic. The point is that a highly open and heterogeneous trading network is a weak buffer against pathogen transmission, infection and contagion.

Live birds are sold at large bustling multi-commodity kitchen markets at the end of numerous Bangladeshi commodity chains: live birds (*broilers, Sonali, spent hens, backyard chickens, ducks, geese, pigeons*, and *quail*), live fish, live cows, live goats, spices, vegetables, fruit, clothes, sunglasses, linen, kitchen utensils, and more. Some markets are divided into sections by commodity type, but poultry shops and other shops are often located side by side with hardly any separation between them. Some markets have designated slaughter areas, while others do the slaughtering and preparation within or close to each poultry shop^5^. Therefore, kitchen markets represent contact zones that lead to particular kinds of entanglement.

Interaction between bird specific commodity chains results in an overall *connected* live bird trading network. Almost all poultry production areas across the country have been shown to be connected through the live bird market network (46). Trading networks form potential routes for viral transmission, including the organization and dynamics of live bird marketing chains via methods by which traders collect and mix birds originating from diverse sources. This generates *heterogeneous* networks in which a few nodes along the trade routes (e.g., markets) may act as hubs, and may promote viral spread (9). Very high virus prevalence in market environments may result from the high level of mixing between birds of different geographical origins and different farming systems (47).

#### Direct and Indirect Trade

*Direct trade* occurs, when a farmer sells birds directly to live bird markets: *market middlemen, wholesalers*, or *retailers*. This constitutes three distinct trading patterns. Direct trade involves 2–4 price mark-ups between farmer and end consumer. *Indirect trade* occurs, when a farmer sells birds to the markets via intermediaries. This is the most common trading method. Indirect trade during normal poultry demand involves first line middlemen and feed dealers^6^. The most common trading patterns, when a farmer sells a particular batch of chickens *during normal demand*, are:

1. Farmer *via* feed dealer *to* 1 or more first line middlemen *to* several markets
2. Farmer *to* 1 or more first line middlemen *to* several markets
3. Farmer *to* feed dealer *to* several markets

For example, if a farmer sells 3,000 chickens, then he may sell 1,000 chickens to three different first line middlemen. The birds are sold at several small and big markets, in the latter to either market middlemen, brokers, wholesalers or retailers. These indirect trading patterns involve 3–7 price mark-ups. In case of *high poultry demand*, then each of the indirect trading patterns may involve additional intermediaries: 1 or more second *line middlemen* or *brokers*. The indirect high demand scenarios involve 4–8 price mark-ups (see Figure 1).

Each trading pattern consists of several variations, depending on the number of traders and market vendors involved in a given bird transaction. Such a trading scenario has implications for the multiplication of market and vendor destinations, likely increasing the risk of pathogen spread, if birds from a specific farmer are infected. Sick birds cannot be traced back to a specific farm. Disease traceability rests on poultry identification and accountability for every live bird consignment between producer and final outlet, which is not in place in Bangladesh.

The farmers make little profit in this scenario. Direct trade would necessitate that they fulfil the role of a business intermediary, contacting buyers and paying for transportation and labour. This would depend on their cash solvency. Most farmers do not have the cash, time and resources to sell their birds directly to markets. Indirect trade through middlemen and feed dealers is therefore, from their own perspective, easier and less stressful.

For example, cash farmer Osman rears 1,000 white broilers. He buys all his input goods in cash from a particular feed dealer. He mostly sells his broilers to markets via this feed dealer, but sometimes also small numbers of birds directly to 3–5 local poultry shops. Direct trade would yield a higher profit, but Osman explains why he prefers indirect trade.

*Osman*: Direct bird trade may give us 2–3 Taka more per kilo, but no one can do this due to the time and effort needed. Indirect trade saves my time, though I make less profit.

Farmer profit depends on the fluctuating prices for day-old chicks, feed and marketable poultry. High production costs can result in lower or negative profit, regardless of the bird selling price. Yet, farmers must accept indirect trade, given their circumstances.

#### Contact Zones Between Farms and Markets

Middlemen employees collect and deliver live birds with changing origin-destination patterns between farms and markets in various districts and sub-districts. Some work for just one middleman, while others work for several middlemen and feed dealers. Their occupational practises may increase the risk of pathogenic transfer. This will be explained by describing the trajectories between farms and markets in the daily working lives of Hanif and Doha: *white broilers*, sourced from sub-districts 20–70 km from Chattogram City, and *spent hens*, sourced from northern districts, 320–400 km from Chattogram City.

On this day, middleman employee Hanif collects 3,000 white broilers from five farms and deliver them to 24 buyers. The five farms are located in sub-districts close to Chattogram City: one farm in Boalkhali, two farms in Patiya and two farms in Anowara. He delivers chickens to four wholesalers and 15 retailers in rural and peri-urban Boalkhali, Patiya and Anowara sub-districts and to four wholesalers at two live bird markets in Chattogram City. He also sells 300 birds to another middleman. This expands the number of final destinations: the other trader mixes Hanif's 300 birds with 2,700 other birds that Hanif's colleague collected from other farms and delivers them to several local and city markets.

Middleman employee Doha is based in Mymensingh, about 360 km from Chattogram City. On the same day as Hanif, Doha collects 1,800 spent hens from four layer farms and delivers them to more than 60 buyers. The four farms are located in three districts north of Dhaka: Mymensingh, Kishoreganj and Gazipur. At night, Doha then delivers them to 10 wholesalers and 50 retailers in 10–12 markets in Dhaka City, Cumilla District, Chattogram City and at a handful of local markets on his way to Chattogram. Such a high number of market destinations remains a business necessity: a wholesaler only buys 100–150 spent hens and a retailer only buys 20–30. In Chattogram City, Doha sells some of his spent hens at the same two markets, where Hanif delivered his broilers.

Such transporter practises exemplify changing contact zones between supplying areas, producers, intermediaries, and outlets, which may increase the risk of pathogen transmission. Changing contact zones include contact zones between separate commodity chains: *spent hens* and *Sonali* from the northern region, *white broilers* from Chattogram Division and *backyard deshi chickens, ducks, geese, pigeons, and quail* from Chattogram and the northern, eastern and southern regions.

#### Contact Zones in Live Bird Markets

The changing contact zone phenomenon continues at the live bird markets between wholesalers and retailers. Chattogram City wholesalers daily sell large volumes of birds to many retailers in several live bird markets. As evidenced, live bird trade *between* markets is common in Bangladesh (46). Price remains the key risk factor for the eventual, but inadvertent, dissemination of infected birds – the lowest buying price, the highest selling price.

Hanif and Doha are just two out of a huge unknown number of people collecting and delivering live birds every day. Upon delivery, Hanif's broilers are mixed with broilers collected by other broiler transporters, at 24 destinations per day. Doha's spent hens are mixed with spent hens collected by other spent hen transporters, at more than 60 destinations per day.

Moreover, different bird types have been shown to be epidemiologically connected through the way they are stored at the markets, whether in the same or separate cages, back-to-back and stacked. Virus prevalence is associated with poultry type. Exchange of viruses within and between bird species can be caused by their direct and indirect mixing at the market level, which promotes cross-species avian influenza transmission (47). Moreover, the prevalence of viruses has been shown to be higher in markets than in farms. Co-infection with high and low pathogenic viruses at market level remains a major concern, since reassortment could produce novel influenza viruses and threaten human health (48).

For example, Ahnaf, a market retailer, trades in white broilers, Sonali and local *deshi* chickens. On this particular day Ahnaf buys birds from 10 middlemen and feed dealers: 900 white broilers from local districts and 100 Sonali from northern districts. He buys 100 chickens from each trader. Each trader bought them at 1–6 farms, depending on price, farm size and truck capacity. In other words, today Ahnaf receives broilers and Sonali from 10 to 60 farms. In addition, Ahnaf takes his rickshaw to buy 150 local *deshi* chickens from three wholesalers at a large commodity trading site^7^ and from two other live bird markets. The wholesalers purchased their birds from several middlemen operating in northern, eastern and southern districts. As Ahnaf says: “*I don't know their farm origins, where the traders buy them.”* But since Ahnaf buys *deshi* from three wholesalers, 150 birds on a particular day may originate from all three regions and an unknown number of backyard farms.

### Patron-Client Relationships

Complex interactions between buyers and sellers characterise the indirect trading, non-market exchange patterns, such as those explored in relation to paddy production and distribution in Bangladesh [e.g., (49)], creating mutual or directed dependence. Such interactions are often managed through non-economic social capital channels of reputation, trust, loyalty and family ties, intrinsically intertwined and matched by mistrust, cheating and negative reciprocity. Paradoxically, such reciprocal give-and-take relation may incorporate “captivity,” in which a farmer is transactionally dependent on a dominant intermediary. A farmer is excluded from receiving the benefits of his efforts in such a situation (50). Such complex interactions may imply epidemiological consequences: unpredictable and meagre profit making among farmers may lead to unsafe practises of last resort, for example the trade in sick or infected birds.

#### Value Appropriation

Credit farmers maintain informal, oral agreements with the feed dealers, promising to sell their marketable poultry to their creditors, thereby paying off their debts. An oral agreement is not a legally binding agreement since it is not written and signed. Exchange is personalised in such informal patron-client relationships between individuals with different degrees of power, commonly seen in, for example, Bangladesh, India and Pakistan (51).

An oral agreement between a feed dealer and a farmer comes with obligations, which are morally, ethically and culturally binding. They trust each other. *Biswas*, the Bengali term for *trust*, pronounced *bisshas*, permeates the retail sector. Written agreements only occur, when doing business with big poultry companies, due to *lack of trust* or company policy. While trust rests on *loyalty, onugatto*, having no written agreement is a particular kind of risk, *jhumki*, pronounced *zhu'ki*, because the feed dealer cannot take legal action, if the farmer is unable to reimburse the outstanding payment.

Traders offer the farmers the lowest price to maximise their own profit. Their decisions and strategies are influenced by the number of intermediaries, each expecting to get a piece of the cake. In Bengali, a patron is a *mahajan*, a big man, a client is a *khuchra*, a small man. Such relationships are intrinsic to Bangladeshi culture, the template mode of social organisation in village communities. In some societies, differences of status associate with the exercise of a limited and diffuse, but none-theless very real power of others Big men appear in places with competitive exchange (52). However, patron-client relationships in Bangladesh, governed by moral norms based on social and religious values and kinship obligations (25), simultaneously imply exploitation and fair treatment: “tie,” “gateway,” and “safety net.” Such dyads constitute reciprocal relationships of rights and expectations: in Bangladesh, higher rank people have the right to extract labour, services and respect from lower rank people. The latter can in turn expect material and other forms of support from their patrons. Such mutual obligation underpins the idea of patron-client relations in this context. However, social institutions such as caste, community, village and extended family, are weak in Bangladesh, compared to other areas of South Asia (53).

Patron-client relationships between feed dealers and farmers in Bangladesh show complex relations of exploitation, opportunity, and risk. For example, Luqmaan is a credit farmer rearing 3,000 white broilers. He buys feed, chicks and medicines on credit from a feed dealer. They maintain an oral credit agreement, relying on trust and loyalty. The feed dealer is cooperative and he always takes quick action upon Luqmaan's requests. Yet, the credit prices are higher than the cash prices and the feed dealer often pays less than the market price for the chickens. This exemplifies patron-client value appropriation through an asymmetric agreement. Any credit incurred must be paid as soon as Luqmaan sells his broilers. It means less profit, when the farmer depends on a business intermediary.

*Luqmaan*: The profit margin is very small to me, as I have to buy and sell everything mostly via the feed dealer. If I had the ability to buy chicks, feed and medicines directly from the market in cash, then definitely I would make much more profit. In that case, I could buy these things at a lower price by bargaining and I could also sell to whoever offered me the best price.

Luqmaan regrets his financial dependency, but on balance he values its advantages. Luqmaan is not a capital owner and therefore incapable of making his own investments. Though his profit is small, it is that small window of opportunity that enables him to at least make a small profit. Credit provides a gateway to remaining in business.

*Luqmaan*: As most of the investment is done by the feed dealer, then sometimes I get 2–3 Taka profit per bird without much more investment. I only pay for the poultry shed and labour. That's why I continue doing business with the feed dealer.

Paying for inputs on credit certainly negatively influences his profit margin, but he has no other choice than to accept this payment method.

*Luqmaan*: If I was the only profit maker, then definitely I would make more profit. But I am poor and I don't have enough money to buy these things in cash. I am actually forced to accept this business deal.

However, a negative economic boomerang effect may happen, when an actor faces economic loss. Other actors will lose money. This may be thought of as *value appropriation rebound*. Creditors engage in *high-risk business*. Feed dealers are themselves subjected to power through relationships with their own patrons – the chick and feed companies. Feed dealers need to adjust their selling price of feed, chicks and medicines between *Trade Price*, TP, from their suppliers, and *Maximum Retail Price*, MRP, to their customers, with an eye to their credit price, in order to keep their farmer customers. Feed dealers may lose money and eventually relinquish parts of their business, e.g., their feed or credit business or ultimately quit as feed dealer. The constant price fluctuations of inputs and outputs affect such circumstances.

For example, Mehedi is a feed dealer and a poultry farmer rearing 8,000 white broilers. He struggles to collect the money farmers owe him from last year, amounting to 2.5 million Taka (30,000 US$). He maintains that the poultry business has become equally difficult for farmers and feed dealers.

*Mehedi*: We have no idea how the production cost of a chick can be up to 100 Taka and how it falls down to 40 and even to 15–20! It is unbelievable! The feed-chick companies are a big syndicate. They set the same price rate altogether. And you have no option to buy from another source. And the price of the mature birds is also illogical.

The fluctuating prices perpetuate a marginal profit trap.

*Mehedi*: It has become an unpredictable business. It really makes no sense to run this business with these huge losses. It is very difficult to believe that the farmers are still surviving. They are hoping that things will change soon, but this will be very difficult.

As a last resort, a feed dealer may choose to stop selling poultry feed on credit. Kashef, a large-scale feed dealer and poultry farmer rearing 3,000 white broilers, chose to stop selling poultry feed by the end of 2016, because farmers were unable to pay for their credit goods, due to disease outbreaks, poultry mortality and a low market price.

*Kashef* : I decided to close my business, because farmers owe me a lot of money that they are unable to pay me back. All my poultry feed business was in due, because of their recurrent losses in their own farming business.

This affected his ability to invest in his key commodities, because all supplying companies only accept cash payments. Now he focuses on cash business: cattle feed, medicines, poultry and day-old chicks. He buys day-old chicks in cash from four companies and therefore always sells the chicks in cash. Kashef fears he would face the same credit crash he experienced from his previous poultry feed business.

The large suppliers want their commodities paid in cash. This requires maximum liquidity. Therefore, a business intermediary runs a big risk, selling goods to farmers on credit. Farmers are often unable to pay their dues on time, because their profit remains unpredictable. In other words, recurrent economic loss among farmers makes a boomerang effect, affecting the lending feed dealers.

#### Trust, Mistrust, and Risk

The survival of credit farmers depends on their ability to settle the informal exchange relationship with the feed dealers. The feed dealer is often the powerful man in the village, at the top of the local hierarchy, but not immune to risk, as exemplified by the value appropriation rebound phenomenon. However, this relationship occupies a contradiction between *trust* and *mistrust* – *biswas* and *obiswas*. “Trust” and “loyalty” were frequent explanations for success in the poultry business, yet informants often referred to their fear of being “cheated” and receiving “fake information.” Cheating, *protarona*, is about deception and trickery, which actors have to keep an eye on in all their transactions. Indeed, it remains imperative to maintain a good reputation. Though cheating is common, it remains a risky practise.

For example, feed dealers may take advantage of the situation to make more profit, at the expense of the farmers. This resembles a patron-client relation more than one based on trust alone. Feed dealer Nadeem explains:

*Nadeem*: I want to share how farmers lose out. Most farmers who buy goods from me are selling their birds through me. They have full trust in me. Sometimes they deliver birds to us according to the rate we demand. They do not always assess the market price and situation. In this way, we can make more profit.

Nadeem aims to understand the daily market situation to ensure informed price negotiation and counter cheating and misinformation by other actors. The fine balance is to navigate the business terrain, aiming for lowest buying price and highest selling price to maximise profit.

*Nadeem*: Look, the experience with negotiating the price of poultry is very complicated. Everyone goes for his own profit. It is a bit tricky. The good side of negotiating the price of poultry is that you can trade by judging, calculating your margin of profit or loss. That gives you the freedom of trade. But there is a bad side to this: you will be cheated by anyone in the transaction process, if you are unaware of the market demand and price situation. You need to handle misinformation tactfully.

In other words, the trade in live birds is a game of strategy. “Fake information” is plausible with rapid and frequent price fluctuations.

Economist Tara Mitchell has exposed the relationship between trust and mistrust: how middlemen are simultaneously exploitative and fulfilling a necessary role in agricultural markets in developing countries. Yet, middlemen dominate the supply chains with substantial market power. An important source of such power stems from the fact that they are better informed about market conditions, the prices further down the supply chain in particular (54). Based on a study of middlemen and farmers in Gujarat, India, Mitchell shows the conflict between two trader behaviours: *fairness* vs. *exploitation* (strategic profit maximisation) and points to the importance of institutional arrangements in the agricultural sector to the welfare of the farmers, which however are all too absent in places, where this is needed.

Accordingly, trust, mistrust and risk are closely related. Trust facilitates decision making and successful transactions. Mistrust leads to a search for defensive mechanisms: “ways to spread risks and weaken dependencies” (55). Cheating could be a risk factor in reciprocal relations, which anthropologist Marshall Sahlins defined in three ways: *balanced, generalised* and *negative*. Balanced reciprocity occurs in gift exchange, trade and buying-selling relations. Generalised reciprocity refers to altruistic transactions, like “assistance given equals assistance received.” However, the idea of negative reciprocity is the most relevant in this analysis, as it refers to cheating, value appropriation and exploitation in unequal trading relations (56).

Farmers may receive less than they expect, influenced by the patron-client combination of trust, loyalty and cheating. Actors mention being cheated in the transaction process as a major risk, yet the terrain inhabits countervailing dishonest and honest traders. In fact, inexperienced farmers fear being cheated, if they engage in direct trade with the market vendors without the protective agency of the skilful, trusted intermediaries. Therefore, farmers show confidence in their intermediaries. Personal trust, through regular business relations, and often family ties, plays an important role in this regard. Yet, such trust may eventually mask the intermediaries' exploitative and cheating behaviours. Ultimately, cheating can be an essential feature and exploitation synonymous with negative reciprocity (56), which may be indicative of the big man-small man relations, apparently intrinsic to, but not limited to, Bangladeshi culture as it exists at this time.

### Precarious Circumstances

In particular, the trade in sick or infected birds involves trust, mistrust and risk. Therefore, in the context of price volatility, patron-client relationships and value appropriation, live bird commodity chains operate under precarious circumstances that may lead to practises that may generate disease situations. We discuss the trade in infected or sick birds as behaviours of *last resort* and of *coercion*.

#### A Behaviour of Last Resort

The farmers themselves raised the issue of unethical behaviour. Indeed, most farmers think it unethical to sell sick birds. The farmers who never do this, refer to Islam: it is considered *Haram*, a prohibited act. This may refer to virtuous ethical life in South Asia, rooted in everyday lived experience (57). But the paradoxical question raised by Veena Das remains: “*… how are we to account for the fact that human beings also act unethically?”* Part of the answer lies in the fact that precarious circumstances may erode the ability to act ethically in everyday life as it unfolds (58). Farmers in dire economic circumstances are forced to breach such religious and cultural principle, adopting such practise as a last resort. The political, economic and policy constraints exposed in the current business environment may foster hazardous practises, such as selling sick birds mixed with healthy birds, which may lead to higher risk of disease transmission and epidemic spread. Selling sick birds remains a secret behaviour. They would face adverse consequences not hiding the information: traders pay less than half the market price for sick birds.

Trade in “sick” birds follows a tendency: It is easier to sell asymptomatically infected birds as “healthy birds,” because it is impossible to identify the infection. It is more difficult to sell visibly sick birds to city markets. Yet, trade in sick birds is ongoing, because a small profit is better than no profit. Visibly sick birds are sold to local shops, local markets, hotels, restaurants and social event agencies on average 40 per cent of the market price. “Apparently healthy birds” are those that are suspected to be infected, as they have been in a flock of visibly sick birds eventually with a degree of mortality.

Middlemen and feed dealers sell sick birds for three reasons: lack of storage and treatment facilities, to maintain good relations with the farmers and to avoid losing money. Farmers have economic reasons for selling sick birds.

Amon, a credit farmer rearing 700 broilers, sells sick birds mixed with the healthy ones to maximise his profit.

*Amon*: I try my best to treat the sick birds before selling. If the birds do not recover and selling time comes, then I sell the sick birds mixed with the healthy birds to the feed dealer. I know it is unethical to sell sick birds, but I have no other choice but to sell them to minimize my economic losses to at least get a little bit of profit.

Rehanul, a credit farmer rearing 2,000 broilers, initially took a 3.3 million Taka bank loan (40,000 US$) to buy land and expand his farm, expected to be repaid within 3 years. Buying his inputs on credit from a feed dealer remains a way for him to continue his business. The fluctuating prices of chicks and feed makes this a difficult challenge. Rehanul previously faced economic loss, so he needs to find solutions to avoid a repetition. He recites a Bengali proverb: “*Father's name will come if I survive.”* This relates to the dictum “self-preservation is the first law of nature.” Then he continues, with a loud, rhetorical voice:

*Rehanul*: When I lose money, how can I think about others' profit or safety! I need to care for my own interests first. I need to minimize my losses! If I have sick birds, then I sell them to the feed dealer mixed with the healthy birds. I know it is unethical and can harm people upon consumption, but I sell sick birds to increase my profit.

Farmers are under pressure to sell sick birds, facing the choices of negative, zero or marginal profit. They have no other choice but to act in their own rather than in the general interest. From their perspective, they act rationally – in economic terms. Reactive behaviours, based on constrained choice, points to actions of *last* or *only hope*. The individual's ability to act independently to escape the determinism of the structural constraints is limited. This is about constrained agency in their everyday farming lives.

#### A Behaviour by Coercion

Yet, credit farmers may also be compelled by their business agreements with feed dealers to sell sick birds. Farmers and feed dealers do not share profit and loss. In other words, they do not share risk in their daily mutual transactions. They do implicitly share risk at a different level, as evidenced in the value appropriation rebound phenomenon, when farmers' inability to repay their debts, leads to economic loss among their creditors. Farmers must repay the feed dealer's loan under any circumstances, including disease outbreaks. In worst case, farmers need to sell their properties, e.g., land, to be able to pay the feed dealers.

For example, Monir's white broilers got sick in 2015 and the mortality was high. A private veterinarian visited Monir's farm and treated the birds, however without any effect. The veterinarian then encouraged Monir to sell his sick birds. Monir was forced to do this:

*Monir*: I tried to sell my sick birds, including the apparently healthy birds, as early as I could, even at a lower price to protect myself from huge loss. I know it is not good practise. But I have to pay for the goods I received on credit from the feed dealer under any circumstances and he never shares losses with me. I was actually forced to do it. If most of my broilers died, then how could I reimburse my credit to the feed dealer?

Monir is mainly concerned about the short-term advantage engaging in otherwise unethical practises, because he has no other choice.

In the absence of regulatory enforcement and compensation schemes, farmers may act in self-interest to maximise their profit. This relates to unsustainable policy. At one point, an avian influenza H5N1 compensation policy, supported by funds from the World Bank, included culling and disposal of affected birds and farmer compensation. This increased the reporting of sick birds. However, the Bangladeshi government was unable to continue this compensation policy, when the World Bank funding ceased. This led to under-reporting of sick birds and likely an increase in the sale of sick birds (29). Selling sick birds becomes a viable option, when the likelihood of punishment is low and in the absence of financial compensation. Indeed, many farmers never sell sick birds, but selling sick birds does become a social survival strategy among farmers forced to challenge ethical principles.

Breaching the forbidden shows resilience and perseverance, but such hazardous practise may inadvertently increase the risk of pathogenic spread. Structure is both enabling and constraining by the inherent relation between structure, agency and power. Given insights from sociology and economics, the choices an economic agent makes depend both on preferences and constraints: *desirable* and *available* decision-making options and outcomes (59). In other words, fewer constraints among the actors would theoretically affect their occupational behaviours positively to eliminate risky practises. Yet, it remains part of the business game to hide information about sick birds and act in self-interest. This is an economic survival strategy. Recovery care would reduce their profit. Such strategy games exemplify self-interested exchange: a self-interested person invariably seeks the choice that maximises personal payoff (60).

## Discussion

This study has examined three key epidemiologically significant risk factors, caused by factors related to agency and structure: *poultry trade, value appropriation* and *the trade in sick or infected birds*. Two key structural factors influence these behaviours: *price volatility* and *patron-client relations*.

Diversified trade in live birds between several commodity chain actors may multiply potential disease transmission pathways. Commodity chains structure contacts between individual birds, influenced by type of trading, number of contact zones, value appropriation and the trade in sick birds.

The trade in sick birds may have an effect on the health of other birds delivered to the markets through different commodity chains. Dissimilar commodity chains divided by bird type interact and connect at the market level (46). Birds from a given farm end up at an unidentifiable number of final outlets, which in turn are connected to other farms, other bird types and other markets. This makes disease traceability impossible.

These behaviours and factors ultimately emerge in a context of precarious circumstances: *uncertainty* and *vulnerability*. The actors play a strategy game, weighing preferences against social, economic and political constraints. Seen anthropologically, such forces constitute contact zones composed by social relations with a potential to host infectious disease *risk hotspots*, representing a convergence of structural circumstances that create such disease communicability (61, 62). Such *risk environment* encompasses risky behaviours, practises and factors – human-animal entanglement, an intermix of bird origins, bird types, people and pathogens. Human-animal entanglements may facilitate the movement of pathogens. Trade in live birds in Bangladesh forms epidemiological networks and such entanglements therefore suggest different viral mixing patterns. Different patterns of contact support different disease dynamics. The configuration of live bird trade may lead to high viral loads in marketed poultry and market environments and this promotes the exposure of humans to zoonotic pathogens (47). Compellingly, entanglement constitutes fundamental characteristics of disease emergence and ecology, which points to critical aspects of biosecurity and complicates the efficacy of control measures, such as contact and disease tracing, biosecurity and trade configuration (45).

We have shown how agency and structure generate a particular risk environment through different forms of entanglement. The question remains, whether the Bangladeshi live bird sector requires a different structural set-up to prevent the risk of avian influenza. Agency essentially means efficient ways to make things happen, but government agency remains ineffective. Prevention of avian influenza – and other animal diseases – is one priority among a huge number of (unfulfilled) priorities in 5-year plans. It is not *top* priority in the Bangladeshi policy environment, compared to other threats and crises (29), referring to diverse needs to society, including education, public health and infrastructure. Meanwhile, individual action remains existential and pragmatic, focussed on immediate short-term gains, eclipsing long-term concerns for the risk and unpredictable spread of infectious pathogens. Given the constraints faced by the government and most agricultural occupations, including the ones that produce and market perishable animal food items, it remains questionable which agent or agents can make things happen to improve the prevention of pathogen transmission to improve public health. Agency and structure point to a much wider issue in Bangladesh. For decades, the appeal to change policy and practise in the agricultural sector has been hampered by a multitude of constraints: political, socioeconomic, material and environmental. Such structural factors reduce agency for both individuals, professions, governments and organisations, pushing pragmatic agency to the fore as individuals seek existential short-term, day-to-day survival. Such risk environment generates epidemiologically significant risk behaviours and factors.

## Data Availability Statement

The datasets presented in this article are not readily available because it is not customary for anthropologists to share raw field data. Requests to access the datasets should be directed to erling.hoeg@lshtm.ac.uk.

## Ethics Statement

The studies involving human participants were reviewed and approved by The Institutional Review Board, Institute of Epidemiology, Disease Control and Research, IEDCR, Dhaka and The Observational Research Ethics Committee, London School of Hygiene and Tropical Medicine. We provided an informed consent statement translated into Bengali, which explained the aims, objectives and affiliations of this study. It promised voluntary participation and confidentiality. It was read aloud for the illiterate. The names of informants used in this paper are pseudonyms to protect their anonymity.

## Author Contributions

Anthropologist EH designed the ethnographic methods and the ethnographic questionnaires and led the field study in collaboration with RM and eight research assistants from Chattogram Veterinary and Animal Sciences University, CVASU. EH was responsible for data analysis, interpretation, and drafting the manuscript. The overall study was originally conceived and designed by TB, DP, GF, and MH under the BALZAC research programme Behavioural Adaptations in Live Poultry Trading and Farming Systems and Zoonoses Control in Bangladesh. TB, GF, DP, MH, and RM critically reviewed the paper, contributed to its intellectual development, and approved the version to be published. All have participated sufficiently in the work to take public responsibility for appropriate portions of the content, agree to be accountable for all aspects of the work, having ensured that questions related to the accuracy, and integrity of any part of the work have been appropriately investigated and resolved.

## Funding

This study was funded from two sources: (1) the BALZAC research programme Behavioural Adaptations in Live Poultry Trading and Farming Systems and Zoonoses Control in Bangladesh, one of 11 programs funded under Zoonoses and Emerging Livestock Systems, ZELS, a joint research initiative between Biotechnology and Biological Sciences Research Council, BBSRC, (Grant No. BB/L018993/1); Defence Science and Technology Laboratory, DSTL; Department for International Development, DFID; Economic and Social Research Council, ESRC; Medical Research Council, MRC and Natural Environment Research Council, NERC. (2) the UKRI GCRF One Health Poultry Hub (Grant No. BB/S011269/1), 1 of 12 interdisciplinary research hubs funded under the UK government's Global Challenges Research Fund Interdisciplinary Research Hub initiative. The funders had no involvement in the conduct of the research or preparation of this paper.

## Conflict of Interest

The authors declare that the research was conducted in the absence of any commercial or financial relationships that could be construed as a potential conflict of interest.

## Publisher's Note

All claims expressed in this article are solely those of the authors and do not necessarily represent those of their affiliated organizations, or those of the publisher, the editors and the reviewers. Any product that may be evaluated in this article, or claim that may be made by its manufacturer, is not guaranteed or endorsed by the publisher.

## Footnotes

^1^Chittagong was renamed by the Bangladeshi government to Chattogram in 2018.

^2^There are two main poultry sectors in Bangladesh in terms of the final product: live poultry and processed poultry. This study focuses on live birds from independent farmers, sold at live bird markets. Live birds from backyard farms, producing local *deshi* chickens and ducks, presents a distinct commodity chain. The *deshi* is the Bangladeshi indigenous chicken, reared in backyard farms in nearly ninety percent of Bangladeshi rural households, each household typically rearing 1–10 birds. As opposed to farmers producing broilers, Sonali and spent hens, backyard farmers take no loans, do not pay any goods on credit and they do not do business with feed dealers. Backyard farmers feed their birds exclusively with domestic resources, they hatch chicks the traditional way, never buying chicks from hatcheries. Moreover, deshi-duck middlemen operate in different ways, compared to broiler-layer-Sonali middlemen. Therefore, the backyard live bird commodity chain needs a separate, independent analysis.

^3^Small-scale farms: <500 birds; medium-scale farms: 500-2,000 birds; large-scale farms: >2,000 birds.

^4^In the commercial live bird sector, all farmers, middlemen, feed dealers, wholesalers and retailers are men and predominantly Muslim. This stands in contrast to the backyard sector, in which most household poultry farmers are women.

^5^A typical live bird shop is about 10 m^2^. The number of poultry shops within a market ranges between 2 and 45 at the 55 identified live bird markets in Chattogram City, a market defined as an open space where at least two peoples sell poultry at least once a week as their main activity.

^6^Feed dealers selling their goods on credit to farmers often provide the business link between farmers and middlemen for free, without making a direct profit. This is a strategy to guarantee farmers sell their birds, since feed dealer profit depends on the farmers' ability to repay their dues at the end of a production cycle.

^7^Chattogram City has two commodity entry points, one for the northern districts and one for the southern and eastern districts, exhibiting classical scenarios of citizens and traders coming to the city with all sorts of goods (e.g., clothes, groceries, live birds).
